# Limb Reconstruction Using the Ilizarov Technique Following Giant Cell Tumour Excision in the Proximal Tibia of a 19-Year-Old Female: A Case Report

**DOI:** 10.7759/cureus.57434

**Published:** 2024-04-01

**Authors:** Virendra E Patil, Sushil Mankar, Pallav Agrawal

**Affiliations:** 1 Orthopedics and Traumatology, N. K. P. Salve Institute of Medical Sciences and Research Centre, Nagpur, IND

**Keywords:** limb lengthening, excision, limb reconstruction, ilizarov, giant cell tumour

## Abstract

Giant cell tumours (GCTs) of the proximal tibia are a relatively uncommon lesion among all benign tumours. They can occur at various sites including distal femur, proximal tibia and distal end radius. Various management modalities of GCTs occurring in the knee joint have been described for reconstruction as well as arthrodesis. We present a case of a 19-year-old adolescent patient with GCT of the proximal tibia with cortical breach with the collapse of the medial articular surface of the tibia. The patient reported experiencing knee pain and swelling for a long duration. Radiological investigations were suggestive of GCT of the proximal tibia with the medial cortical breach and collapse of the medial tibial articular surface. The patient was managed with a resection followed by arthrodesis using intramedullary nails with bone grafting, followed by Ilizarov reconstruction due to osteomyelitis of the surgical site.

When dealing with relatively aggressive tumours that have breached the cortex, wide resection of the tumour is required. Following this, the reconstruction procedure must ensure good biomechanical tenacity, biological healing, infection resistance, and intact function of the knee joint extension. One option for achieving this is total knee replacement with a customized prosthesis, though this can be costly. Another option is joint arthrodesis with intramedullary nailing or the Ilizarov fixator, which is strongly supported by the existing literature. This case was managed successfully with the above-described method, and complete healing was observed. In conclusion, periarticular long bone tumours, especially around the knee joint, can be managed effectively with the Ilizarov method. Though it has a few disadvantages, such as a long duration of external fixator, non-compliance, and pin tract infections, it still stands as a viable alternative for limb reconstruction due to its cost-effectiveness and time-tested efficacy.

## Introduction

Giant cell tumours (GCTs) are a type of bone tumour that can be locally aggressive [1]. They can range from benign to locally aggressive and may even have malignant potential in some cases [2]. These tumours are most commonly seen in young adults between the ages of 20 and 40, with a higher incidence in females [3]. GCTs are typically found in the epiphyseal-metaphyseal region of bone (more than 80% of the cases)and are often located within 1 cm of the sub-articular bone. The knee joint, both the distal femur and proximal tibia, as well as the radius at its distal end, sacrum and the proximal end of humerus are some of the most common locations where these tumours are observed [4].

GCTs that are localized, with no cortical breach or involvement of soft tissue masses, typically respond well to extended curettage. However, for advanced-stage tumours (Campanacci grade III), wide resection is often necessary.

The treatment is usually surgical curettage with a main focus on preventing recurrence, as there is a high local recurrence rate observed postoperatively [5]. For patients having extensive destruction of the joints, especially weight-bearing joints like the knee joint, excision along with reconstruction of the knee joint is promoted as the incidence of local recurrence and a good knee range of movements is achieved giving the patient a better quality of life [6]. Reconstruction with endoprosthesis sustains a high cost of treatment, multiple surgeries, and aggressive long-term rehabilitation [7]. The short-term results seem promising but currently, there is less evidence of long-term outcomes of prosthetic treatment [8]. On the other hand, the other treatment option like arthrodesis of the knee joint seems less appealing, but holds several advantages like less chance of repeated surgeries, providing a stable painless joint, and less chances of recurrence, significantly low cost of treatment [9]. There are very few documented cases of Ilizarov-assisted knee arthrodesis following the destruction of the knee joint following a GCT.

We report a case of a 19-year-old female patient who suffered from a proximal tibia GCT, managed with resection, followed by failed knee arthrodesis, and finally knee arthrodesis limb reconstruction with Ilizarov.

## Case presentation

Patient information

A young 19-year-old female patient came to our orthopaedic department having complaints of pain and swelling observed around the right knee joint for eight months. The pain was present just distal to the right knee joint and the patient noticed it increasing with time. Initially, the knee movements were unaffected until the flexion gradually decreased with increasing size of the swelling.

Clinical findings

The patient was thoroughly examined clinically. The patient was unable to bear weight over the right lower limb. On examination, a bony hard swelling around 5 cm by 6 cm was seen on the anteromedial aspect just distal to the right knee joint. There were no signs of inflammation and the skin over the swelling was shiny but normal. There was no distal neurovascular deficit.

Diagnostic assessment

**Figure 1:**
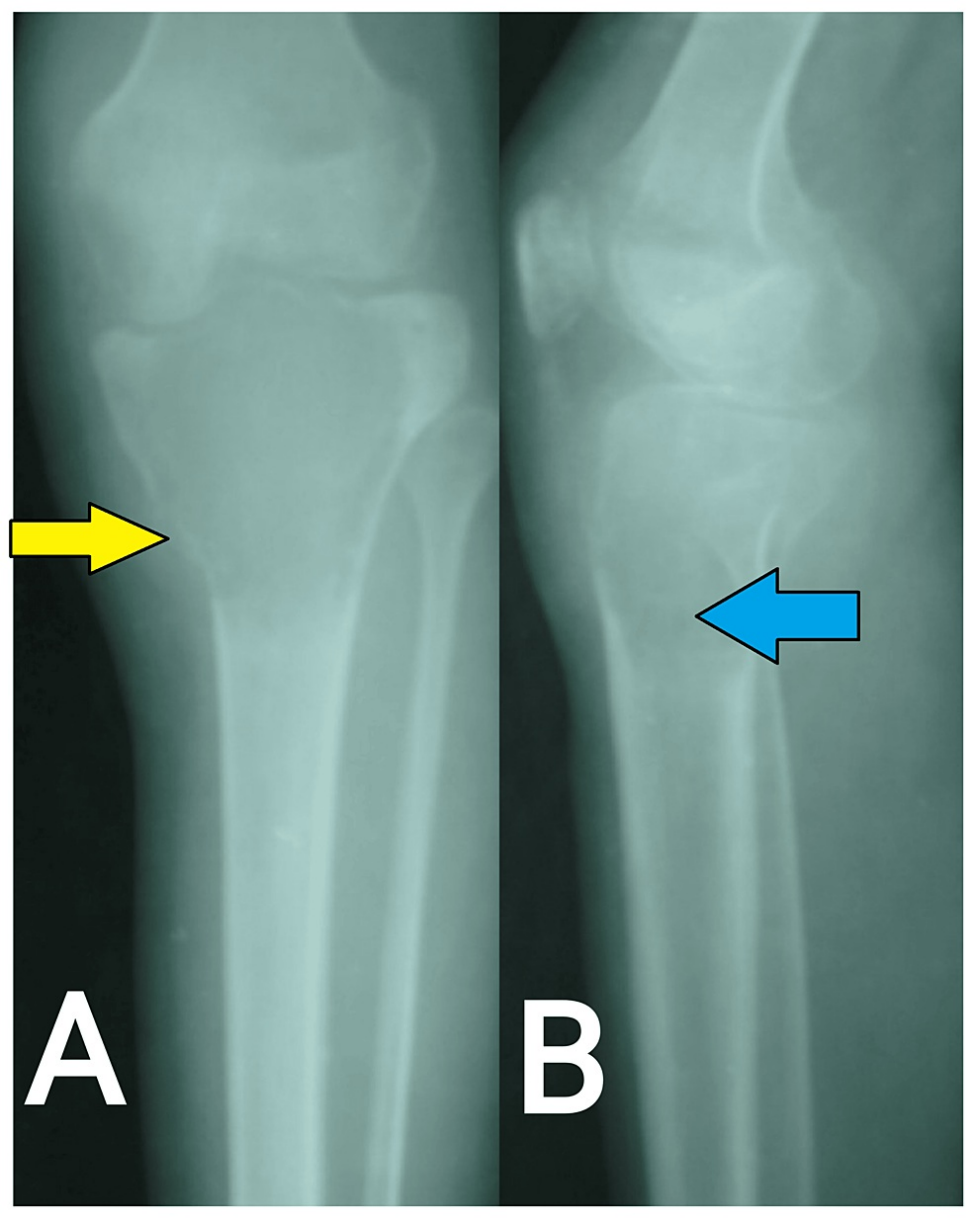
A plain radiograph of the right knee with the proximal tibia (anteroposterior and lateral views) displaying a lytic lesion over the proximal end of tibia with thinned-out cortices. (A) Anteroposterior view showing radiolucency and thinned-out medial cortex (yellow arrow); (B) Lateral view showing radiolucent area (blue arrow).

**Figure 2:**
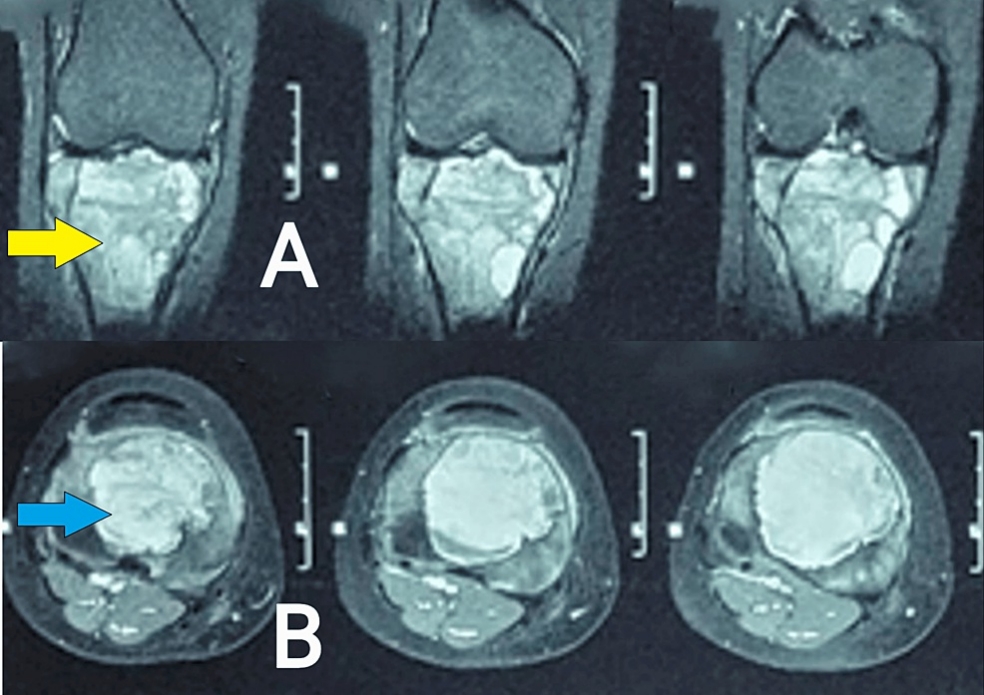
MRI images showing axial and coronal section of the right knee joint with the proximal tibia. (A) Coronal section showing an expansile lytic lesion (yellow arrow); (B) Axial section showing a lytic lesion (blue arrow).

**Figure 3:**
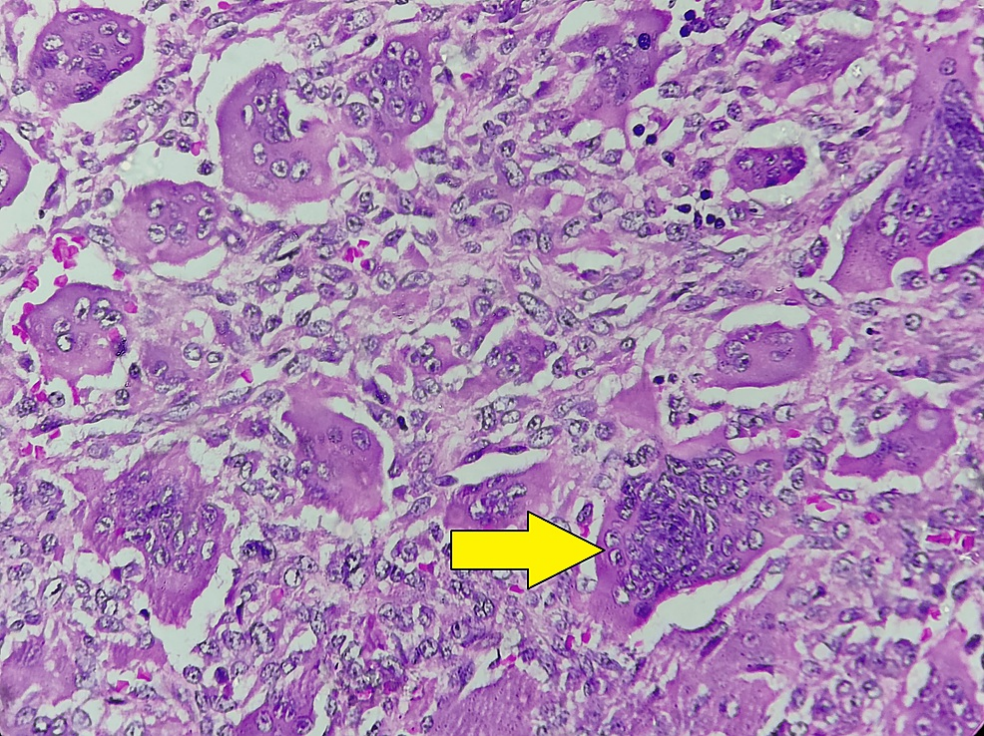
Histopathology examination Large multinucleated osteoclast-like cells in cluster (yellow arrow)

After the clinical examination, the necessary haematological and radiological examinations were done. The haematological examination revealed mild anaemia with no other significant findings. Plain radiographs of the knee in both anteroposterior and mediolateral views were done which displayed a large expansile lytic appearing lesion over the proximal end of tibia with thinned articular cortex and medial cortex (Figure 1). There was no breach in any of the cortices but the mild depression of the medial tibial condyle or other pathological fractures which were suggestive of a GCT. For further investigation, an MRI of the right knee joint and proximal tibia was done to confirm the X-ray findings (Figure 2) which showed a well-defined heterogeneous lobulated expansile lesion measuring 4.6x5.1x6.3cm in the metaphysis of the tibia with medial cortical breach. Further investigations, including chest X-rays and a CT scan of the abdomen, were done to rule out lesions at other locations. Biopsy was also done which revealed round polygonal mononuclear cells with multinucleated osteoclasts like giant cells (Figure 3).

Surgical planning

After confirming the diagnosis patient was given the option of salvaging the knee joint and giving her a pain-free mobile joint with custom total knee arthroplasty. But due to financial constraints, the patient was planned for excision with arthrodesis using a tibiofemoral nail with bone grafting.

Surgical procedure

**Figure 4:**
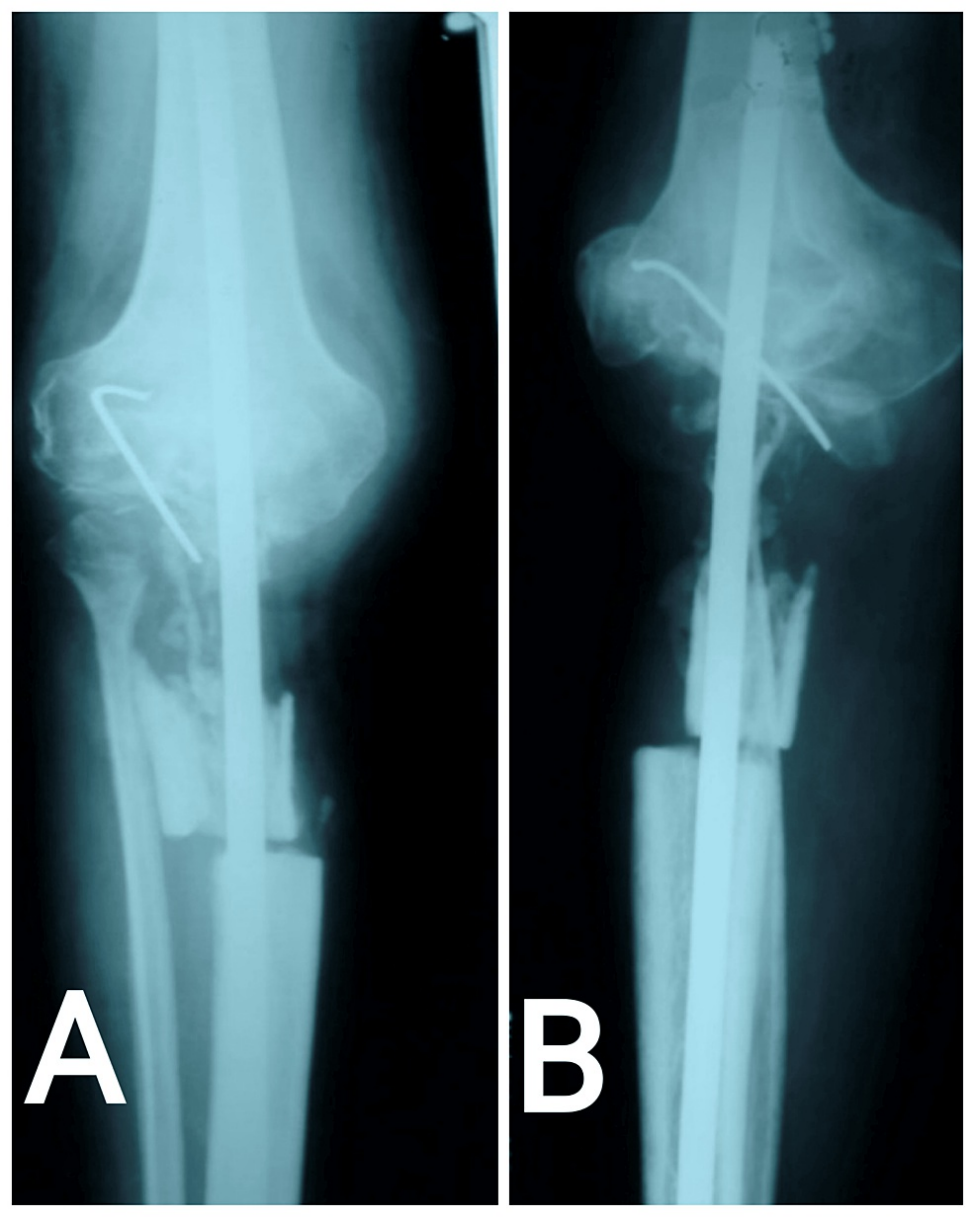
X-ray showing resection with bone grafting and arthrodesis with tibiofemoral nail. (A) Anteroposterior view; (B) Lateral view

After thorough planning and with all necessary informed and written consents, the patient was operated on with resection arthrodesis with a tibiofemoral nail with autologous bone grafting. The resected specimen was sent for further investigations to confirm the diagnosis. Post-operatively radiological X-rays were done (Figure 4). Our patient was allowed partial weight bearing on the limb as much as tolerable by her with a gradual shift towards full weight-bearing mobilization. The patient was recovering at an acceptable rate.

**Figure 5:**
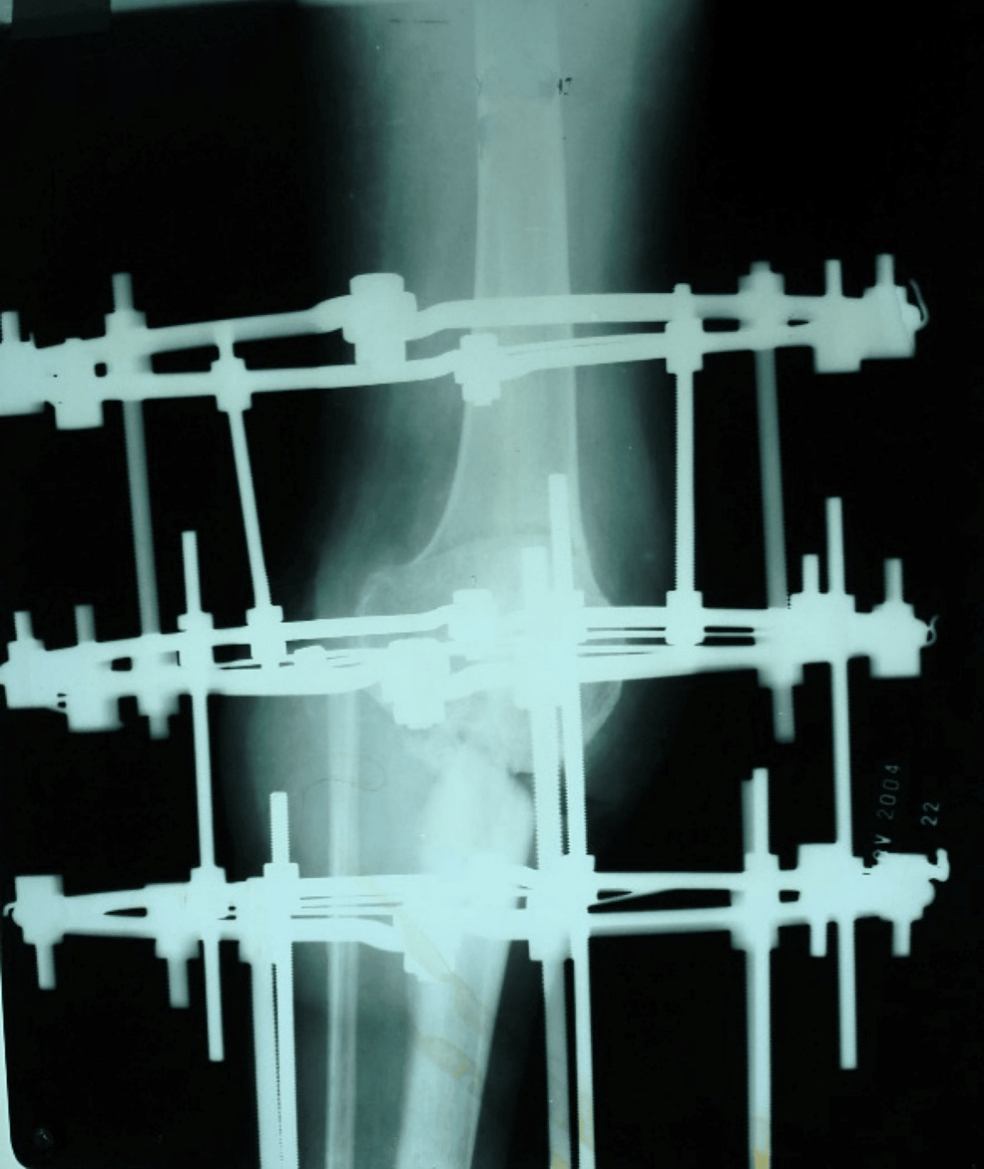
X-ray of a knee joint showing Ilizarov ring fixator with distal femur corticotomy for lengthening.

After two years of uneventful period, the patient started complaining of pain and pus discharge from the right knee joint (surgical site) with painful weight bearing. Haematological investigations confirmed infection at the surgical site. The patient was explained and then operated on for surgical site debridement, bone block removal and limb reconstruction using the Ilizarov technique (Figure 5).

**Figure 6:**
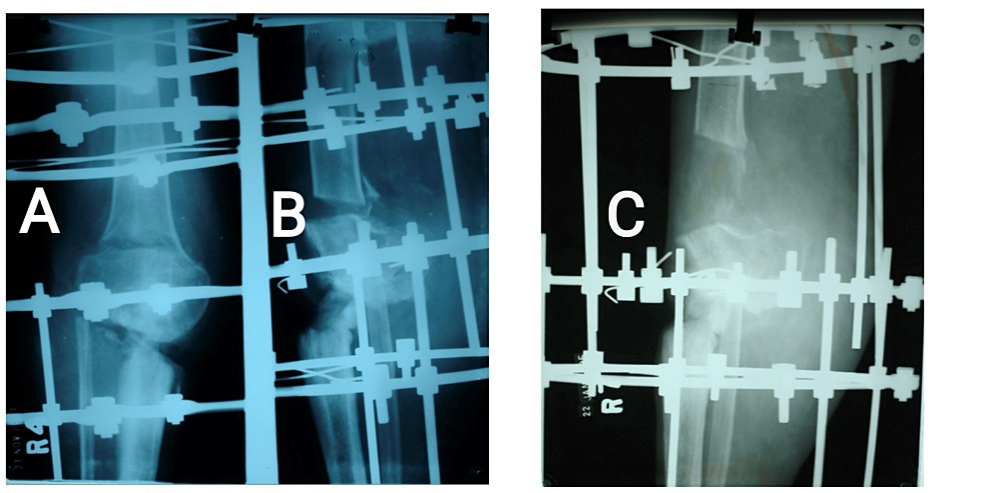
Sequential X-rays showing initial distraction followed by completed lengthening at the corticotomy site. (A) Anteroposterior view; (B) Lateral view both after initial distraction (one week); (C) Lateral view of X-ray after complete lengthening (after 10 weeks).

Docking at the debridement site was done that caused a shortening of around 7 cm, which was addressed by lengthening by gradual distraction at the distal femur site (Figure 6).

Follow-ups and outcomes

**Figure 7:**
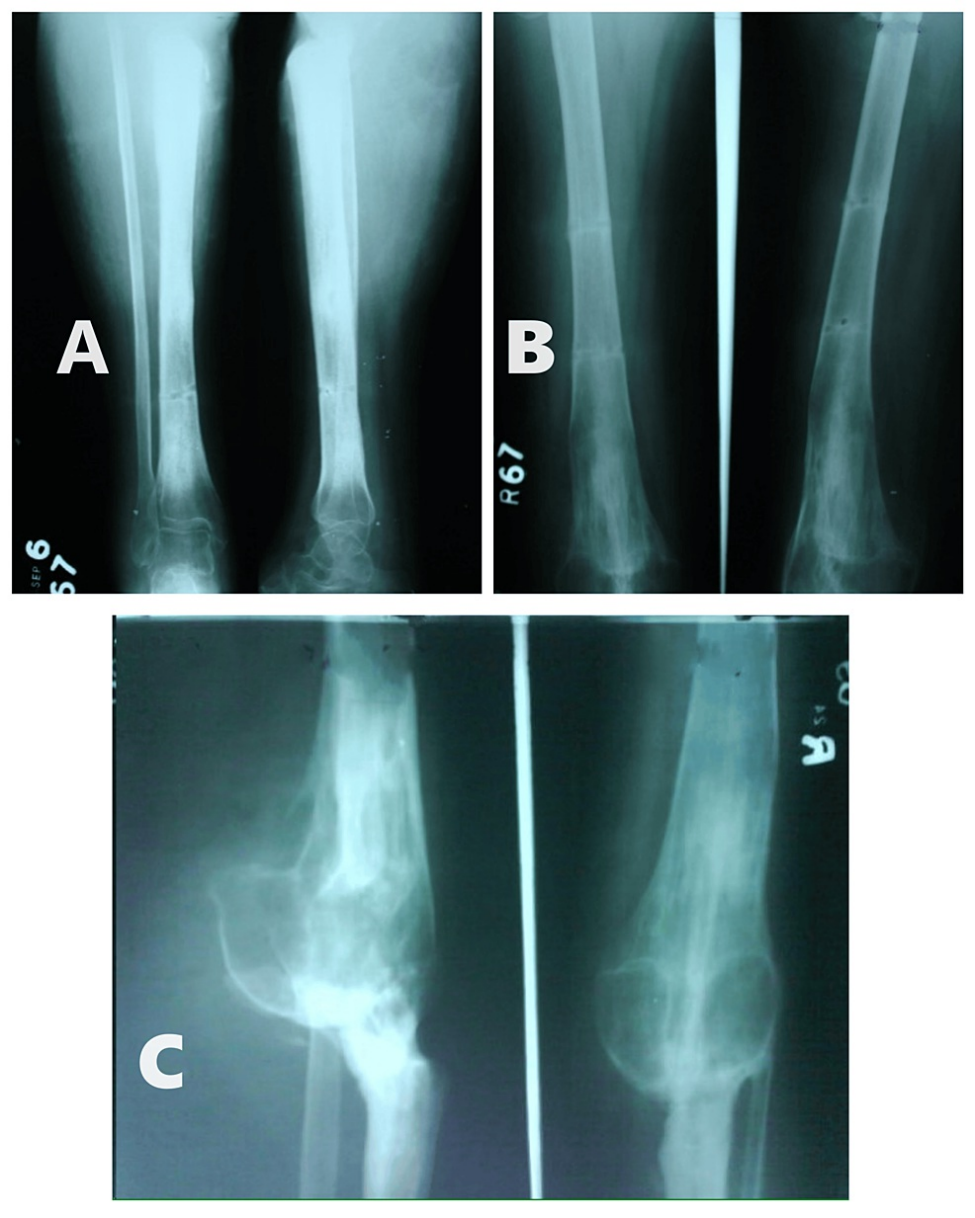
X-rays showing complete union at the non-union and distraction site. (A) Anteroposterior and lateral view of tibia and ankle joint; (B) Anteroposterior and lateral view of femur shaft; (C) Anteroposterior and lateral view of knee joint.

**Figure 8:**
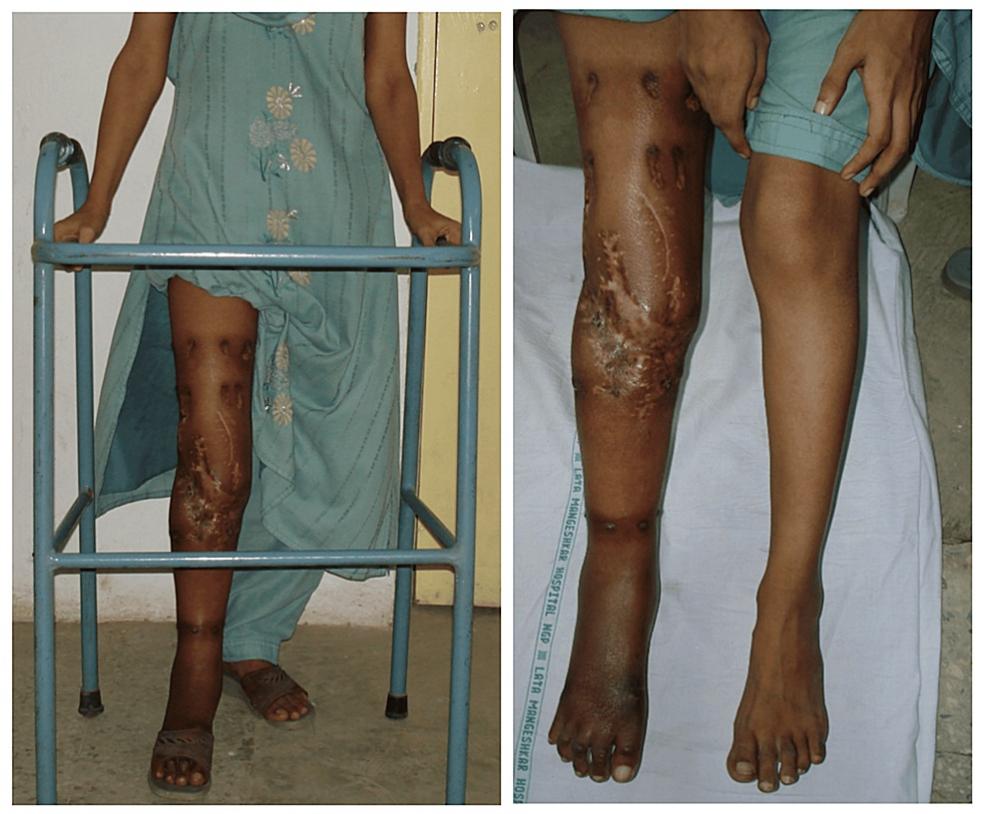
Clinical images of the patient showing corrected limb lengthening with no signs of infection.

Distraction was continued for a period of 10 weeks with 6cm of distraction and the total duration of frame was 40 weeks to complete union at the non-union site. Serial X-rays were done to confirm the consolidation of the distraction site, union of the bone at the docking site and to correct the limb length discrepancy. Finally, the frame was removed after 40 weeks after confirming the radiological and clinical union at both the non-union site as well as the distraction site. Post-removal X-rays were done to confirm the union and the lengthening (Figure 7). The patient was given a thermoplastic full-length brace to aid in walking and was allowed partial weight-bearing walker support walking for a period of four weeks thereafter full weight-bearing walking without any aid (Figure 8).

## Discussion

GCTs around the knee joint are relatively rare. Surgery is the main treatment to reduce the risk of recurrence [10]. The desired management demands a good balance between ample removal and limb function restoration [11].

Options in surgery include curettage followed by chemical cauterization, bone grafting or cryosurgery-aided extended curettage with bone grafting, and wide resection with appropriate reconstruction [12]. Subarticular cases with the cortical breach require wide resection with reconstruction of the defect with various methods. The reconstructive method for large bony and soft tissue defects generated after wide resection of aggressive GCTs is aimed at limb salvage along with a stable pain-free mobile joint, especially with tumours around the knee joint [13]. Post-excision of GCT from the subarticular area, reconstruction should provide adequate biomechanical durability, infection resistance, biological healing and keeping intact function of extension of the knee. This is achieved by the total replacement of the knee joint done with a custom prosthesis [14]. This surgical method comes with a huge cost, longer rehabilitation and chances of infection. There is ample documentation on the option of joint arthrodesis with intramedullary nailing or with an Ilizarov fixator that can be used successfully in many cases [15].

Management of our case was successfully done by the above-described method and complete biological healing was observed.

## Conclusions

In conclusion, periarticular long bone tumours, especially around the knee joint, can be effectively managed with the Ilizarov method but it only comes with the disadvantages of prolonged duration of the external fixator application, non-compliance, pin insertion site infections, and low social acceptance. However, arthrodesis using the Ilizarov method is a viable alternative that offers a long-lasting and economical tool to reconstruct limbs.
